# Sexuality and hierarchical trajectories in Global South: A de-colonial reading of Sefi Atta's novel, *A Bit of Difference* in the exemplification of contemporary literature

**DOI:** 10.1016/j.heliyon.2022.e10159

**Published:** 2022-08-10

**Authors:** Christopher Babatunde Ogunyemi

**Affiliations:** Institute for Gender Studies, University of South Africa, Pretoria, South Africa

**Keywords:** Women marginalization, Development, Sexualities and feminism, Decoloniality, Contemporary literature. global south

## Abstract

A study is made to the created gender dichotomies and the creation of transnational mobility shift in Nigeria's Sefi Atta's novel, *A Bit of Difference* (2012). Using the novel and related trends, the article seemingly valorizes the dialectics of transnational migration on gender identities in Africa. It examines the critical discourses of feminist configurations, migrations, sexualities in the Global South countries. It constructively examines the consequences of dexterity and trajectory of gender and sexual realities and migration tenets on the cultural matrix of the people. Apparently, the article visualizes the societal tropes of peoples of the global South. It further probes into the obtrusively constructed identities and transnational migration underscoring its attendant consequences on women. It demonstrated how these impeded the exponential development of the Global South or developing countries. Suffice to say, masculine and feminist harmonization have greatly been subjected to transnational endangerment and religious strangulations aiding divergent migration of women. The article foregrounds the coloniality of gender and the polemics of male streaming of the patriarchal power. In these developing nations under examination, women and some queer functionality have not adequately attained the proper attention to advance the plethora and dynamic life events. This have constantly opposedthe polarized and escalated representations of gender proximities in divergent medium of feminization of migration processes. Together with diffuse societal configurations of gender, this article delineatesthe notions of transnational migration and sexualities, hierarchies and the trajectory of women oppression. It also examines the corollary of male genderizations in different obvious speculations. It is a backdrop against sexual prejudice and identity polarization in Global South. The article x-rays decolonial lens and Butler's performativity to re-enact the reconstruction of gender ‘performativity’ and cultural foregrounding ‘beyond the coloniality of gender’ impasse. This will facilitate the development of critical reasoning, social realities and dovetail into the development of men and contextualization of the liberation of the genderized males and females.

## 1 Introduction

Sexuality and hierarchal trajectories in Global South have been largely demonstrated in both current and extant literatures. This informs the need for decolonial theory to the explication of gender and violence in African literature. Women and men have responded positively and negatively to these scenarios by either migrating or face some dire consequences which showed the various levels of inequality they suffer. Transnational migration and mobility among women and men in Africa have enormously been ascribed to a contextualized societal reality with the aid of development of socio-economic strata in society. Though, more men were assumed to migrate more than women, this could be predicated on the high inequality of economic power in women in most Global South societies. However, in order to enhance economic growth, political and social development, human capital flight among the Global South has been actualized. This has encouraged a plethora of gender violence and distinction. Consequently, ‘gender based variations of daily mobility is an established phenomenon in both the developed and developing parts of the world’ (Uteng, 4). These gender distinctions and violence therefore have constituted some onus in critical works of art in the functionality of gender diversity in Africa.

The epistemological functionality of gender is predicated on the multiple and diverse complex-complications of concepts ignominiously revolving around colonial structures. These colonial structures have grossly encouraged the inequalities experienced in frequent migration of men, women and children particularly in some Global South countries. For example, much skilled and unskilled labor migrated from Nigeria, Ghana, and Cameroun to South Africa in recent years. Colonial structures embodying transnational mobility, trans-culturalism, ethnocentrism and radical polarity have ostensibly constituted some of the mainstream of thoughts which pervert indigenous matrix among the Global South and other developing nations. In contemporary writings, Abraham opines that ‘gender generates public interests and postulates scientific and neuroscientific postulations’ (Abraham, 2). Moreso, Fausto-Sterling axiomatically asserts that ‘from the fields of phenomenology, dyadic interaction, gender provides insights into inter subjectivity and the emergence of subjective identity’ (Fausto-Sterling, 2) make the study of sexualities and identities contemporary and complimentary while examining the matrix of transnational mobility and migration on movement motifs in Global South.

The trajectory of colonial embodiments on gender praxis seemingly impeded indigenous thoughts and order leading to transnational mobility and transcultural endangerment. The coloniality of gender and incessant women subjugation are some of the perennial postcolonial foregrounding tenets craving for divergence dichotomies. It also materializes into subservience been experienced among some women when they encounter their male counterparts in the course of their mobility. Similarly, Cunha (2005) and Uteng (2012) all portend that Global South or developing countries could transcend toward greater dimension of development if evolved considerably through equality, empowerment and focused gender lines that unearth transformation and contextualized opportunities. Similarly, Uteng (2012) quoting from Cunha (2005) opines that:

[A] recap of the development interventions undertaken in the developing countries to impact genderequality, women's empowerment and poverty reduction highlight their limited success. Despite extensive discourse and resources that have focused on women as key actors for development, their situation has not changed considerably. A little unearthing reveals that regardless of using gender as a label, most policies and programmes failed to truly incorporate gendered issues primarily due to a lack of understanding of the contextual realities and a dilution in the process of transforming goals to implementable projects (Cunha, 4).

The position of women as typified and exemplified by Cunha (2005) on transnational mobility and migration contextualized the cultural dichotomies structured by the existing colonial and post-colonial functionalities in most developing countries. Literatures emanating from most African countries, particularly in Nigeria and South Africa, Kenya and Uganda have demonstrated the rhetoric of vulnerability of women at the expense of men. Furthermore, such literatures have also delineated the polarization of genderized males at the hands of strong males. Similarly, literatures arising from seemingly women stereotyping areas of Brazil, India, Saudi Arabia have dovetailed into the matrix of're-colonization’ of women. In Saudi Arabia, for example the impact of religion and the concept of women tolerance have constituted critical discourses. Apparent distinctions between men and women, cultural allocations and the creation of designated identities among men and women have enormously and grossly endangered the teleology of the existence of women in these areas.

This article, therefore, displays the link between the sexuality and hierarchy which exists in the African purvey. It visualizes the decolonial propensities and queries the epistemology of transnational migration around men and women. It showcases the representations of colonial and post-colonial enquiries on gender configuration in developing areas. It factors the temerity behind the purported exponential transnational migration and mobility arising from the dialectics of overt gender prejudice. It phantoms the notion that decolonial lens and ‘performativity’ in Butler's intensity could tremendously enhance the position of women beyond the mere matrix of bi-gender. The article interrogates a plethora of related events in African cosmology which re-enacts the configuration of gender and migration. It demonstrates how societal factors like religion and inequalities underscore migration. For examples, recurring issues like, inequality among young girls in Arab African countries and Saudi Arabia evoke the tenets of gender inequalities been perverted on women in limiting them to exercise freedom of movement. The overarching trends in cultural and psychological disengagement among men and women in a cosmopolitan society is a factor. This, in most cases is dominantly motivated by overt religious conflagration in most African countries like Somalia, Ethiopia and Sudan. In addition, the article will show the divergence andescalation of attendant catastrophic attack on women and their subjectivities cut across the Global South which hinder women during migration unlike their male counterpart.

## 2 Theoretical framework: decolonial theory and Butler's ‘performativity’ in gender consciousness towards transnational migration consciousness

Decolonization paradigmatically expands the horizon of human consciousness in different dimensions. It explicitly and syntagmatically interrogates the stream of fixated interpretations of cultural ethos leading to new awareness arts and culture. Decolonization enables the proper understanding of societal fundamentals. Most especially, overt gender and identity display, sexuality disposition, migration and mobility. It also helps in the understanding of migration and mobility of men and women in a smaller scale and in the larger dimension. For so long the existentialism of gender in different critical consciousness has been considered a colonial enterprise because of the way it fashions the allocation of roles and cultural sensibilities in societies for men and women. While colonialism offers an ontological acceptance to the existing norms of subjection and subjugation, decoloniality as buttressed by Mignolo and Escobar (2009) offers a critical consciousness and sensibilities that is predicated on individual dependency. They further underscore colonization as ‘the darker side of modernity’. Acording to Heleta, this leaves them to define decolonization as ‘the epistemological restitutions that exist with political and ethical implication’. Heleta (3) views this as ‘a way to re-orientate people away from colonial supremacy’ which would encompass ‘ontological resistance and enhance radical transformation among people’ (p. 4).

Decoloniality is not totally rejecting foreign values but re-constructing these values to be internalized, relevant and accommodating in divergent world cosmologies. According to Benhari-Leaks (2019: 68), decolonial enquiries is a post-colonial shift that underscores the link between pedagogy of knowledge, epistemology of identity, relevance of culture and the stream of consciousness. Some of the stream of consciousness could encapsulate migration and gender preoccupations as well. To this effect, it is a form of ‘decolonizing knowledge *which*necessitates shifting the geography of reason to beyond Eurocentric and provincial horizons and producing knowledge beyond strict disciplinary impositions. However, the decolonizing movement aims to make explicit the links between political and economic *framework*. Decolonizing knowledge is not simply about de-Westernization or rejecting Western streams, nor is it about closing doors to European or other traditions’ (Behari-Leaks 66, Moya 18 and Nakata 12). Similar proections were illuminated in the works of Moya (2011) and Nakata (2007).

In an attempt to re-construct gender and transnational migration and link decolonization to cultural hegemony and relevance, which examines gender and mobility of both men and women in society, Butler (2005) interrogates the discourses of sex, identity and hierarchal beyond mere reductionist theories. She identifies different complexities of the projection of gender praxis beyond mere ‘heterosexual matrix’ (Butler, 19). According to her ‘this situation further explains the ‘unproblematic unity’ and in Butler (1993: 16) it evokes the ‘solidarity of identity’ in the areas of 'sex and identity’ (Butler, 2005: 24). She axiomatically argues that in most situations women are seen as ‘materialist’ when conditioned as a subordinate. She further opined that instead of this assumption, there is need for ‘another order of materiality’ (p. 26). Therefore, in the bid to escape and seek a better life, women sometimes escape the huddles imposed on them by the male dominated society. Example of this could be seen in the notion why women of Southern Cameroun, South Sudan and Congo migrate to South Africa where human right is at its highest peak.

There could be a resistance or implication, the implication could be that sometimes males use language to ‘construct gender and identity so that they could satisfy their heterosexual bodies’ and other representations (ibid). This sometimes inform class disposition men and women sometimes experience at work place or during the process of transnational migration. Butler assumes that different behavioral dispositions could be enhanced at any time to justify that gender is acted and it is a performance in totality. Low income women and psychologically disposed women were sometimes affected by this behavioral disposition. This performance to Butler could be reversed vice versa and also rehearsed in society. In *Gender Trouble* (11), she opined copiously that to be heterosexual or homosexual, obviously, is ordinarily a state of the mental condition in which person may find himself or herself embedded. Therefore, she demonstrates the performativity of gender's diverse and multiple contradictions. This would re-construct human perception in order to fight for the right of the oppressed in society. When we fight the obnoxiously created hierarchies and identities there would be transformation for both females and males in egalitarian society. Such created metaphors or identities/sexualities do not transform society that is a reflection of socio-economic development. Socio-economic change is also a vital factor which transcends gender based migration among the Global South in most cases.

In most cases more men tend to migrate and leave women and children behind sometimes because of the societal norms and believes which rest responsibilities on men. Moreso, a dynamic society should not encompass created frameworks which promote gender and class egalitarian principles. In addition, such society should not illuminate some certain hierarchic configurations that are preoccupied and vested in interests in biased. ‘Performativity’ as a cultural framework in the words of Butler entails the notion that the woman would not want themselves to be identified with those ignominiously created artificial creations of the heterosexual projections because they are not performative. Those claims, according to her, are mere heterosexual enactments handed over to them from generation to generation. Therefore, it is now imperative and exigent to change them so as to be *performative* and for the mainstream of development for women and men to be shaped by diversity that is devoid of dominance in society.

Similarly, some of these experiences of hierarchal, patriarchal and sexual discrimination are imminent in some Global South nations because of the existing customs and inherent norms. These customs and norms were sometimes explained differntly. According to the United Nations research (2007) where 'the most educated young men in Kosovo were paid higher than their female counterparts'. This has encouraged the concept of attributing low and inferiority disposition or language by some phallic writers to women and children. Invariably consummated, such assignment of inferior language to women, and in other development, the assignment of superior instinct or master tone to men was obvious in extant literatures of those areas. This inferiority representation of women and children may spark the need for relocation to neighboring countries. Consequent upon that, Butler's *Gender Trouble* (10) frowns at such portrayal as opprobrious. She opines that ‘language of usurpation suggests a participation in the very categories which s/he feels inevitably distanced, suggesting also the denaturized and fluid possibilities of such categories once they are no longer linked causally or expressively a fixity of sex’ (p.10). This valorizes the distinct differences which occur in the way men and women use of language which sometimes depict the application of language in a syntagmatic or in paradigmatic way in society. To explain further, the paradigmatic relationship exists within the level of signifier and/or signified, or both, but not at the same time, syntagmatic use of language demonstrates a sequential relationship which exist between two (Saussure 1959:11).

To buttress the concept of decoloniality in Sefi Atta's A Bit of Difference and to avoid mere generalizations, the perception of Butler helps stimulate the performance of theories and traditions in the specific understanding of gender and violence. In Exciting Speech: A Politics of the Performative Butler discourages the language use which does not demonstrate relevant and performative elements in society. Such language, sometimes include ‘injurious speech’ which is demeaning and fixated. By so doing a discursive conversation between females and males are supposed to enhance harmony. To Butler someone (either female or male) is simply not permanently fixed through the notion and such person is being addressed. The implication here is that, if some persons or someoneis being called an injurious name, s/he could be limited, demeaned and derogated psychologically, physically and socially. In that situation, the name addressed to such individual do not holds out the necessary possibility.

Butler, however, believes that such injurious address may appear dangerous and inhuman. Such an utterance is located within the framework of a “total speech situation” (Butler, 1997: 4). This means also that ‘there is no easy way in which one can decide on how best to delimit that totality of utterance in society’ (ibid, Butler, 4). Therefore, for a proper articulation of ideas in society, gender is more projected as a cultural perspective which should be seen from that framework instead of illicit allocation of dangerous languages, roles and assumptions which are not culturally motivated.

## 3 Sexuality and migration in the Global South

In Nigeria, Saudi Arabia, Sudan and Kenya, to mention a few, the discourse on sexualities, hierarchal representations and gender identities has constituted the canons and the overarching experience of the Global South. Other global trends on discourse include religious tenacity, constant quest for women liberation and women hindrances. The similarities of history around women and men writers have historically trapped the dexterity of the miasma of hapless representations of women in literature and society. These women, in most situations are truncated by obnoxious trans-cultural waves, transcultural migration which lead to struggle for equal rights, contextualized identity configuration and hierarchal perversion. For this reason, the selected novel, which is a typification of gender based and transnational migration fiction would re-examine women vulnerability beyond the onus of coloniality. It would examine the role of gender and strategically view how the homonormative Western mainstream of consciousness have led to many contradictions women experience in one location or the other.

Sefi Atta's *A Bit of* Difference (2012) is a novel that encapsulates overt trends in cultural disposition. It exerts preoccupations with allusion to trans-cultural migration and its attendant effects on gender parity among men and women.

Briefly examining Attah's Biography, She was born in Lagos, Nigeria. Sefi Attah is prominent in creative writing; she is an author and a playwright. Her thematic preoccupations encapsulate contemporary social matrix. Other concerns she titillates include gender and feminist motits, underdevelopment and corruption in African literature. She was educated in Nigeria, England and the United States. In her novel, the representations of women and experience shift from one locale to the other. Consequently, Attah's novel, according to Ogunyemi (2020) ‘is also a constructive response to women displacement and dispossession’ in Nigeria. Ogunyemi, (2020: Fela Kuti's Black Consciousness, 2). The plot of the novel revolves around Deola Bello, the female protagonist. Most feminist novels would rather feel comfortable creating a female protagonist who would saturate the plot from the beginning, middle and end. During the plot movement, the intricacies of migration for women and phallocentric reactions were usually captivated. The female protagonist in A Bit of Difference relocates to London to work and enjoy good life. She later relocates to Lagos to seek marriage and employment. There is a shift from Global South to Global North and back to Global South. This is the implication of transnational gender based migration. The complexities and polarities of cultures become shifted from London to Lagos. These migration complexities are constantly hindered by external factors she experienced as a result of her female nature. The London urban landscape craves asymmetrically for creativity and avid characteristic interplay is exchanged for the Lagos radiating cultural approach. This intersects both social and religious temerities underpinned by family and friends' influential-consciousness. That is why Uteng remarks that ‘it is necessary to consider the social realm as a setting for everyday life with respect to national legislation, cultural conduct and societal practices’ (p. 46).

The novel opens with the migration possibility, its effects and consequences on Deola. It dovetailed into the notion that Deola the female protagonist was over thirty years and still very single. During the earlier experience as buttered in the novel, families want her back home to seek a man who understands the African way of life. Though a very agile woman, strong and mobile, she needs the presence of a man in her life as expected by her African way of life. This belief is opprobrious and obnoxious considering the present dispensation where humans are treated equally and with high respect. This concept or believes are sometimes peddled in some Global South countries like Nigeria, Ghana and other African countries. Her marital status becomes a topic of discussion among her mother and her siblings. Even with the death of her father, she had to come to Lagos to see the possibility of getting married to a waiting suitor.

Many people relocate from Global South to Global North to escape obnoxious gender parity and enslavement. This form of relocation and other thematic constructions in the perception of Ogunyemi (2020b) have ‘become the foundation for widespread poverty among women because they have been vigorously exposed to greater risks among peers and different societal cultures’ in their bids for survival (Ogunyemi 2020b, *Contextualizing the Versification*, 5). In the long run find them incapacitated with the huddles of Global North and escape back to Global South where they come from initially. The portrayal of women as communal property and issue of discussion preoccupied the corpus of the novel. In London, the reverse is the case. The spate of childlessness or unmarried state does not concern anybody. The life in Nigeria is communal, such like other African countries. In the United Kingdom, life projection is individualistic and personal. The decision to marry or remain unmarried is of no concern to nobody's interest. To Butler, the situation of depression on women for whichever reason is ‘problematic’ and ‘injurious’ when women are oppressed and forced to migration against her will. To her, gender is a mere creation of ‘sexed bodies’. These gendered bodies can be expressed through which ever means. Since ‘gender mirrors sex’ therefore, there is an obstacle to this connection which is one creates by the individualised in a society. She opines critically that the illusive display of gender in society regardless of its limits is a performative application which makes it fictional and dynamic.

Butler in totality rejects the notion of oppression in exchange for new epistemologies which best explain identity configuration and gender. The protagonist is well informed of that. That is why in Butler's discussions of various categorizations of gender, sexualities, hierarchal projections and sex praxes, she prefers the ‘performative’ aspect rather than ‘the illusory’ aspect, which most extant literatures profess. Such illusory assumptions which Deola experienced in *A Bit of Difference* (2012) is fondly conceived in the works of Luce Irigaray and Simone de Beauvoir's view of women in art. In *Bodies That Matter*, Butler re-echoes the perception that, ‘if gender is a construction, must there be an ‘I’ or a ‘We’ who enacts or performs that construction? She further posits that ‘how can there be an activity, a constructing, without presupposing an agent who precedes and performs that activity? How would we account for the motivation and direction of construction without such a subject?’ She further opined that, ‘if gender is constructed, it is not necessarily constructed by an ‘I’ or ‘We’ which stands before it in any spatial or temporal sense’ (Butler, 7). The creation of gender polarity and other constructions assigned women from their state of unmarried and childlessness are illuminated by some people in Lagos in the novel. The novel through thematic preoccupations and other devices have in Ogunyemi (2018) ‘helped in the establishment of a new twist and tradition for women development in society’ (Ogunyemi 2018, *Gender (re-Configuration*, 129)).

Deola Bello's original family root is deeply foregrounded in Islamic religion. Many women have relocated from the huddles of Islamic religion to a more stable place and egalitarian society like South Africa that is a religion free setting. Islamic religion, in most cases accommodates the African patriarchy and entrenches its fundamental principles. Women suffer in most cases as reflected in pages 4, 9 11 and 19 of the novel. This informs the reason why El Saadawi (2006) remarked that in all ramifications women must reject both patriliniality and patriarchy. The admonition was to sensitise the Egyptian women, African women and, indeed all women in the Global South. Similarly, Okafor (2002) opined that these inhabitations have limited women spaces in societies. Deola, the woman protagonist has a plethora of records of these influences of religious tenacity on her space as she leaves and arrives in Lagos, Nigeria.

In the novel, characters syntagmatically follow tenaciously Muslim culture to the letter. This informs the reason while when still single, the mother is worried and compares her to all her siblings who are fully married. Concurrently, sexualities and hierarchal projections are the trajectories which she had to contend with when she comes in contact with her fellow Nigerians and other African contemporaries. She had to contend with the victim of racism in London, particularly at her work place. This makes her a victimof migration in London. Though, highly educated. In the novel, she possesses the fundamental ability and the possibilities of exchanging pleasantry with both men and women that come in contact with her. To substantiate this assertion, she once remarked that ‘the war is different. Everyone she knows in London is outraged. Everyone wants to win the debate, which has become a separate war. Strangers are co-opting her as an ally, including a drunken man who was seated next to her on the tube’ *(A Bit of Difference*, 19). It is ironical that such situation may not easily happen in Nigeria because of incessant class polarity, obnoxious self-importance and unbridled glorification of status arising from demeaning the low class persons.

The drastic shift to transnational migration in Global North experienced the degree of self-realization and purposefulness which is overtly displaced by Deola in London. She enhanced independently the sense of place and power. That is why she related well with Kate and the teleology of womanhood was accurately exemplified by the duo who occasionally related positively in a symbiosis dimension. This migration shift enlarged incessant depression and melancholy and was frequently experienced by Deola. She does not really believe in herself. She is an epitome of that isolated individual African woman in Diaspora who is truncated by African communal polarity and displacement.

The notion of her being single or unmarried were constantly been mentioned. This portend a psychological fear and inhibit her actions when in contact with fellow Africans. Although she is financially independent and slightly melancholic, she tries to empty her mind from these embellishments by actualizing some invisible and individualistic tenets. This scenario to Butler is an ‘attribute which facilitates the field of discourse and power that orchestrates, delineates and sustains that which qualifies human activities’ (Butler 1993:7–8).

Furthermore, *A Bit of Difference* (Attah 2012) probes distinctively into divergent dissonances of life. It dovetails into different life arousing from Global South to Global North and back to Global South in a revolving parlance. The created metaphysical themes of women and migration, the metaphors of life their consequences illuminate the epistemology of deconstruction on transnational migration. In a detailed presentation, Sefi Atta re-invigorates the perceived inordinate conceptions of Nigerian men as untouchable and sacrosanct. They are perceived as ‘sacred cows’ who could date any woman, indulge in infidelity and multiple sex relations with or without the cognizance of their wives. While these go on, they use both the Christian and Islamic fundamental instruments as the opium of the people without being checked or controlled.

There is a huge family demand ranging from cultural obedience and abstinence from taboos created by men. Such taboos, such as marriage delay and suitor's rejection are to be avoided by young women which make them seek marriages in distant lands in some cases. The ambivalence of men's disposition is an equivocation that lands most women in trouble and rejection. This underscores the dominance of men in most Nigerian society and culture which the novel encapsulates.

Similarly, the fiction portrayed critically the secrets behaviors of men and their gay lives which they concealed from families, friends and colleagues. Some men and women in West Africa, similarly, relocate to South Africa where gay life can be practiced without inhibition. In Nigeria, anybody practicing gay life is sentenced to fourteen years' imprisonment without sympathy. Similarly, other Global South nations like Zimbabwe could not accommodate gay men and women. However, India and Pakistan could not contain some freedom for women in the realization of their spaces. Saudi Arabia recently allowed women to drive cars and there is a limitation. It is also similar to different levels of consternation and trepidation meted out by some men to women in the novel. This is an exercise of power by men which incessantly comes with resistance from women. That is why Foucault asserts in his submission that ‘there are no relations of power without resistance’ (Foucault 1981, 141). He visualizes resistance in human being as an internally existing phenomenon which is a consequence of something that reacts against that which exists as a result of the construction of power.

But for Butler in this relation of ‘power and resistance’, she submits that Foucault's feminist vision exists beyond the premise of identity and politics. According to her, ‘Foucault's fragmentation within feminism is a paradoxical opposition to feminism from ‘women’ whom feminism claims to represent. It is also a necessary limit to identity and politics' (Butler 1990: 4–5). Thus, Butler views Foucault's feminist's epistemologies as a source of strength in a more advanced and dynamic exploration of the feminist fundamentals. This is because Foucault's assumptions sensitively provide new ideas and possibilities in the representation of the issue of sexualities, hierarchy, identity and to the heterosexual bodies. Foucault's projections of the notion of identity with regards to the individual help feminism in the prioritization of the political thoughts which depict divergent representations of types of stereotyping of sexes.

The obvert characterization of sex and identity by Foucault does not necessarily mean that such is in the real sense artificially and arbitrary. Summarily, Butler assumes that the Foucauldian epistemology on the construction of power and resistance with the categorization of identity creates a configuration of diversities of meanings which will enable more pluralized understanding of the place of one in a dynamic society which explain the fundamental framework behind transnational migration and gender mobility.

## 4 Conclusion

The article critically examines the dexterity and trajectory of gender proximities on transnational migration in the Global South. It re-defines the configuration of gender dichotomies on women and their attendant consequences. Religious and social strangulations have been enormously adduced as the fundamental factors inhibiting the performance nature of women in most Global South countries, particularly in Africa. The article probes into the recurring decolonial and Butler's performativity to proffer the exigency to appreciate factors in its original state and relevance. Though gender is a cultural phenomenon, it inexplicable connotations are merely and intricately acted in society. The article demonstrates that transnational migration is within the whims and caprices of both men and women. Women are highly disadvantaged in the process because of lot of intelligible and ineligible factors which hinder the migration of women from one locale to the other. Sefi Attah's novel, *A Bit of* Difference (Attah: 2012: 16-18) therefore, 'reveals the obtrusive economic migration and transnational cultural impasse women suffer' in a drastic conglomerate shift from Global South to Global North and back to Global South. The article attempts to ameliorate the recherché references to the obnoxious concept of women monolithic representations as a result of inordinately created dichotomies in society.

## Declarations

### Author contribution statement

All authors listed have significantly contributed to the development and the writing of this article.

### Funding statement

This work was supported by University of South Africa.

### Data availability statement

Data will be made available on request.

### Declaration of interests statement

The authors declare no conflict of interest.

### Additional information

No additional information is available for this paper.
